# Optimizing Dam Detection in Large Areas: A Hybrid RF-YOLOv11 Framework with Candidate Area Delineation

**DOI:** 10.3390/s25175507

**Published:** 2025-09-04

**Authors:** Chenyao Qu, Yifei Liu, Zhimin Wu, Wei Wang

**Affiliations:** School of Geosciences and Info-Physics, Central South University, Changsha 410083, China; chenyaoqu@csu.edu.cn (C.Q.); yifeiliu@csu.edu.cn (Y.L.); wuzhimin@csu.edu.cn (Z.W.)

**Keywords:** dam database, YOLOv11, random forest, multisource remote sensing

## Abstract

As critical infrastructure for flood control and disaster mitigation, the completeness of a dam spatial database directly impacts regional emergency disaster response. However, existing dam data in some developing countries suffer from severe gaps and outdated information, particularly concerning small- and medium-sized dams, hindering rapid response during disasters. There is an urgent need to improve the physical dam database and implement dynamic monitoring. Yet, current remote sensing identification methods face limitations, including a lack of diverse dam samples, limited analysis of geographical factors, and low efficiency in full-image processing, making it difficult to efficiently enhance dam databases. To address these issues, this study proposes a dam extraction framework integrating comprehensive geographical factor analysis with deep learning detection, validated in Sindh Province, Pakistan. Firstly, multiple geographical factors were fused using the Random Forest algorithm to generate a dam existence probability map. High-probability candidate areas were delineated using dynamic threshold segmentation (precision: 0.90, recall: 0.76, AUC: 0.86). Subsequently, OpenStreetMap (OSM) water body data excluded non-dam potential areas, further narrowing the candidate areas. Finally, a dam image dataset was constructed to train a dam identification model based on YOLOv11, achieving an mAP50 of 0.85. This trained model was then applied to high-resolution remote sensing imagery of the candidate areas for precise identification. Ultimately, 16 previously unrecorded small and medium-sized dams were identified in Sindh Province, enhancing its dam location database. Experiments demonstrate that this method, through the synergistic optimization of geographical constraints and deep learning, significantly improves the efficiency and reliability of dam identification. It provides high-precision data support for dam disaster emergency response and water resource management, exhibiting strong practical utility and regional scalability.

## 1. Introduction

Dams serve as critical infrastructure for flood control, irrigation, and power generation [1,2]. They underpin local agricultural production and domestic water supply while simultaneously representing physical structures susceptible to disasters. Their safety and the accuracy of their spatial distribution data directly influence regional disaster prevention capabilities. However, existing global dam spatial databases exhibit significant limitations: as of 2020, the GOODD (Global Georeferenced Database of Dams) [3] documents approximately 38,000 dams worldwide with geographic coordinates. Constructed through manual digitization using high-resolution Google Earth satellite imagery, it achieves a spatial accuracy of 0.3 m and represents the first global spatial database encompassing small- to medium-sized earth–rock dams. While it provides dam wall coordinates and associated watershed vector files, it lacks detailed attribute information and relies on manual annotation for updates. The GRanD (Global Reservoir and Dam database) [4] suffers from update cycles exceeding five years in Asia and covers only medium to large dams exceeding 15 m in height. Consequently, numerous small dams vital for agricultural irrigation (constituting over 60% of provincial totals) remain unaccounted for. FHReD (Future Hydropower Reservoirs and Dams) [5] catalogs approximately 3700 planned or under-construction hydropower dams, emphasizing new projects in developing regions (e.g., South Asia, Africa). It includes project phases, projected reservoir capacities, and preliminary environmental impact assessments. However, it lacks precise geographic coordinates, experiences infrequent data updates reflecting project progress, and fails to integrate some completed projects into GRanD or GOODD. GDW (Global Dam Watch) [6] integrates the aforementioned three databases with the Global River Obstruction Database (GROD). Version 1.0 contains 41,145 obstruction locations and 35,295 associated reservoir polygons, representing 7420 km^3^ of cumulative storage capacity and 304,600 km^2^ of artificial water surface area. Despite this, spatial data gaps for small- to medium-sized dams persist in developing regions. Clearly, omissions of small- to medium-sized dams and delayed updates remain critical gaps in addressing disaster risks. Large-scale global monitoring struggles to meet the demands for fine-scale management and timeliness in localized dam administration. Considering these factors, there is an urgent need for fine-scale, rapidly updated, and intelligent technologies targeting smaller regions. Such technologies are essential for constructing comprehensive local dam spatial databases to support regional disaster emergency response.

In recent years, high-resolution satellite imagery has provided a new approach for dam identification and monitoring [7]. Remote sensing imagery can deliver real-time, large-scale ground information, aiding in the rapid determination of disaster-affected entities and impacted areas [8,9,10]. This capability helps reduce the time and cost associated with manual surveys while providing timely data [11]. Consequently, dam identification and extraction based on high-resolution remote sensing imagery and spatial constraints contribute to accurately delineating disaster risk zones and establishing disaster entity databases. This approach facilitates ongoing monitoring of potential hazards, holding significant implications for earthquake prevention and disaster relief.

Current research on dam monitoring and identification in remote sensing primarily differs in two aspects: (1) global discrimination methods utilizing geographic features to form spatial constraints [12] and (2) regional discrimination methods employing full-scene scanning. The former approach considers that dam locations often exhibit specific geographic characteristics. These geographic features, defined as points, lines, or areas, or categorized based on the underlying logical relationships associated with dam presence, provide spatial constraints on potential dam locations [13]. Zhiyuan (2019) [14] utilized an automated lake/reservoir extraction method combining an adaptive bimodal threshold to define regions of interest, subsequently extracting dams within candidate areas using a neural network feature model trained with the Faster R-CNN algorithm. Wang et al. (2024) [15] quantified the influence of topographic factors, hydrological factors, and the Dam Detection Ratio Method (DDRM) on dam presence. They constructed spatial constraint strategies using these factors and compared the identification performance when integrated with four deep learning models: YOLOv5, VariFocalNet, Faster R-CNN, and Cascade R-CNN. Ran (2015) [16] improved water body segmentation methods to identify bridge dams but did not differentiate between the two structures. The extraction of these geographic features employs object-based classification and extraction methods; they are capable of accurately extracting texture and geometric information from remote sensing imagery but appear highly image-dependent. Relying solely on these geographic features for global dam extraction often significantly compromises model accuracy and identification precision, while failing to provide precise geographic information.

In contrast to the above methods that extract geographic information, build models, and perform target recognition on the entire image, some researchers opt to divide the full image into smaller tiles for individual scanning and target dam identification when detecting dams across large areas. Jing et al. (2021) [17], recognizing that while the YOLO model is fast and accurate, it might still miss some dams in large-area detection, adopted a sliding window detection strategy. They used an initial dam position fusion method based on Non-Maximum Suppression (NMS) and length thresholds to identify overall features. Yafei (2022) [18] addressed the size inconsistency between large-format, high-resolution remote sensing images and deep learning object detection models by employing a sliding window cropping method. This preprocesses the large images into standard image patches, identifies dams within these patches, restores their spatial coordinates, and completes dam detection combined with the YOLOv5s-ViT-BiFPN model. Zeyu et al. (2022) [19], tackling the challenge of large image formats and small target dam sizes, used a water index-constrained sliding stride method combined with an improved YOLO algorithm (E-YOLO) for large-format water conservancy facility detection, analyzing the accuracy differences between large and small stride recognition. This sliding window approach consistently scans for dams tile-by-tile. However, applying small windows sequentially over large areas demands substantial computational power. Processing imagery at this scale inevitably consumes significant resources and costs, making it more suitable for dam-dense regions prone to frequent floods or with extensive hydraulic infrastructure. Yet, predicting the density of dams in a specific area before identification and detection is difficult, potentially increasing ineffective workload.

To address these challenges, this study aims to comprehensively analyze various geographic elements surrounding dam locations. Leveraging high-resolution remote sensing imagery, we employ a Random Forest model to identify candidate dam areas through integrated geographic element assessment. Within these high-probability areas, a YOLOv11-based detection model is established to construct a high-coverage dam distribution dataset. This dataset serves as a reference for local authorities in disaster response and contingency planning.

## 2. Data and Methods

### 2.1. Study Area

**Figure 1:**
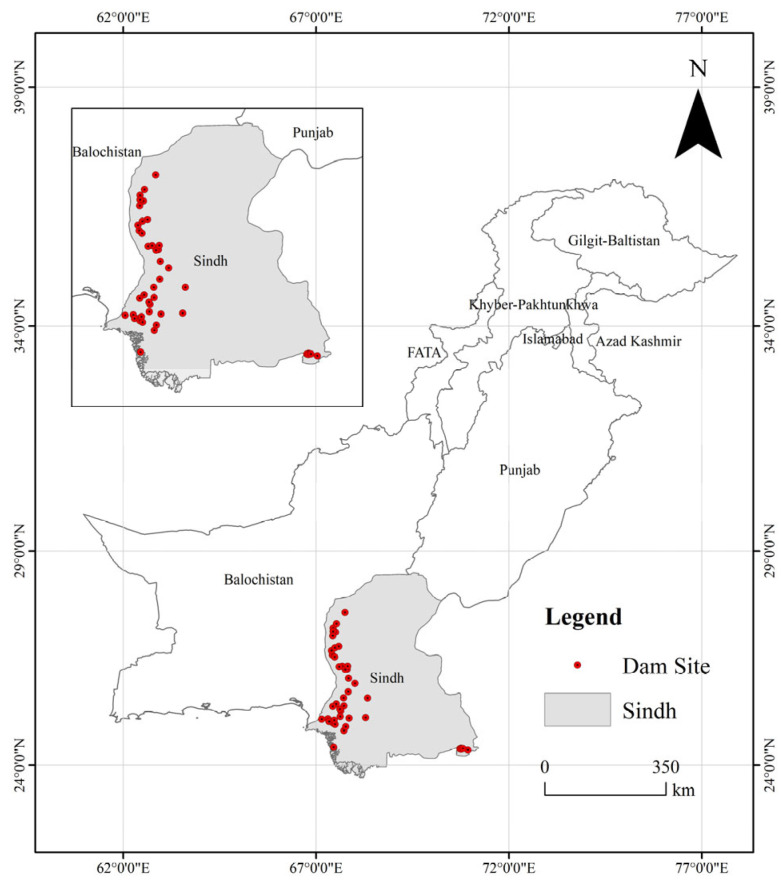
Overview of the study area in Sindh Region, Pakistan.

The study area encompasses Sindh Province, Pakistan (24°37′ N–28°25′ N, 66°03′ E–71°06′ E), as shown in Figure 1. Located in southeastern Pakistan, the province extends southward to the Arabian Sea and covers an area of 140,900 km^2^, accounting for 17.8% of the country’s landmass. It lies within the lower Indus River plain, characterized primarily by flat river valleys and an arid, low-rainfall tropical climate. The Indus River flows 412 km through the province, with 78% of its discharge regulated by 23 control structures, including the Lloyd Barrage and Ghulam Muhammad Dam. According to the 2022 World Bank Assessment Report, these facilities support a provincial irrigation network covering 53,000 km^2^ (38% of cultivated land), safeguarding 43% of Pakistan’s cotton and 31% of its rice production. However, compounded by downstream channel sedimentation (averaging 1.2 cm/year) and extreme climate events, Sindh ranks among the world’s regions at highest flood risk. The 2010 megaflood breached 37 provincial embankments, inundating an area of 7579 km^2^. The 2022 flood event further triggered piping incidents at 12 major dams along the lower Indus, directly endangering 2.3 million people [20].

### 2.2. Data Sources and Pre-Processing

#### 2.2.1. Terrain Data

**Figure 2:**
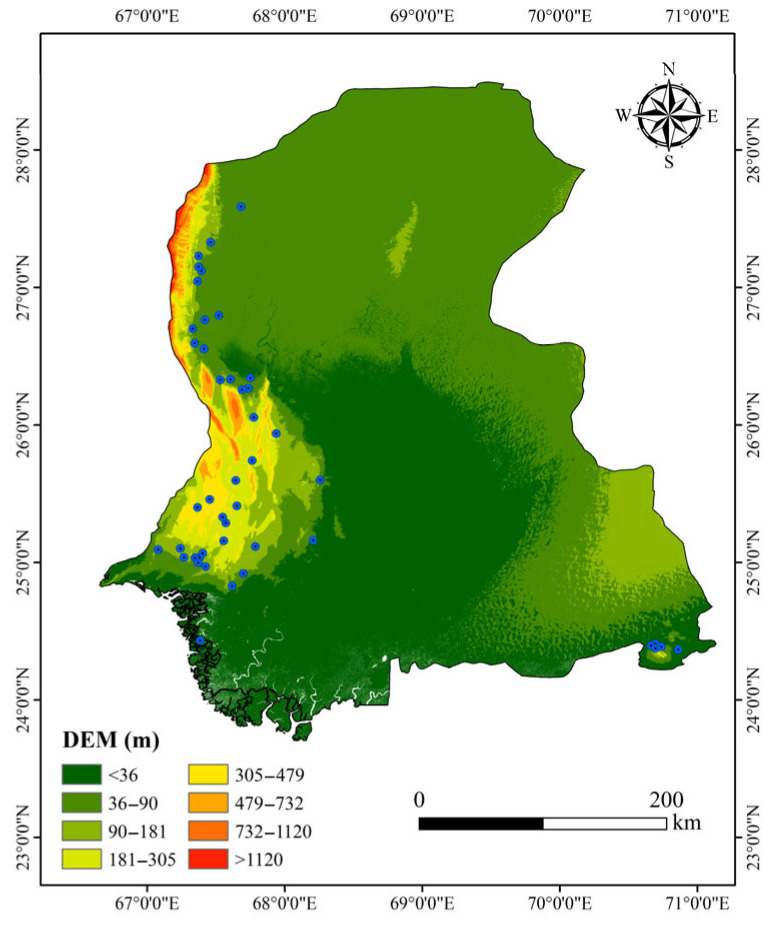
DEM of Sindh Region.

**Figure 3:**
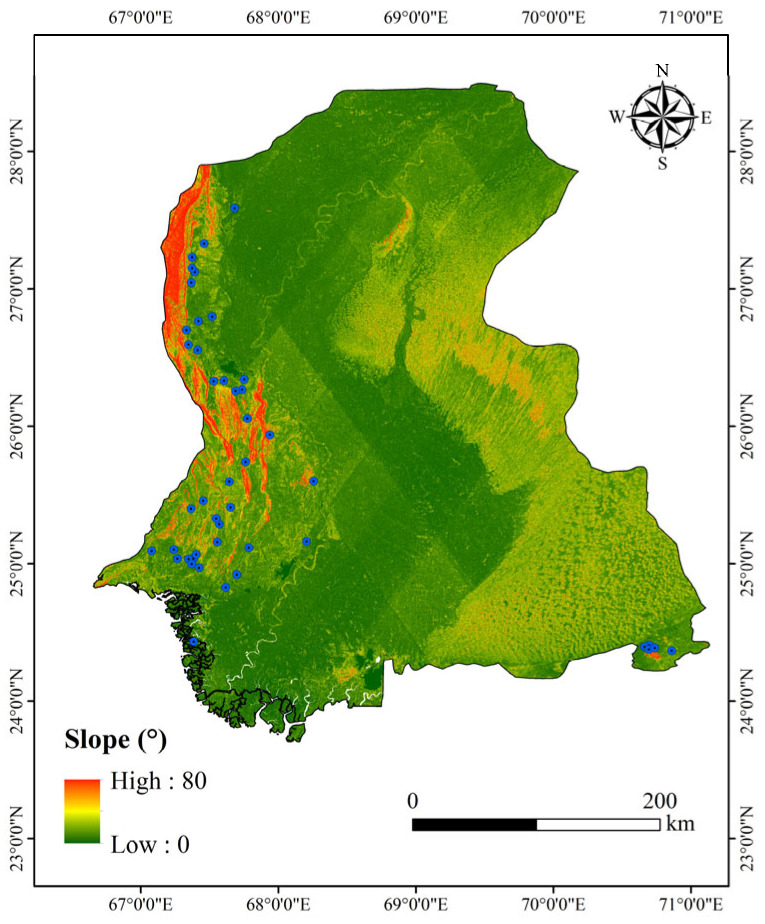
Slope of Sindh Region.

Terrain characteristic data were obtained from the 1-arc second (approximately 30 m) resolution Digital Elevation Model (DEM) of the Shuttle Radar Topography Mission Version 3 (SRTM V3), released by the National Aeronautics and Space Administration (NASA), as shown in Figure 2. This publicly available dataset, with a vertical root mean square error (RMSE) of less than 10 m [21], represents one of the medium-to-high accuracy terrain data sources. It was used to extract terrain factors including Slope and the Terrain Ruggedness Index (TRI), employing the following methods:

Dam foundations should be located in areas with gentle slopes (5–15°) and stable rock strata. Slope was calculated using the second-order finite difference method to construct a dam foundation stability classification model, as shown in Figure 3.

(1) $$S_{suit}=\left\{\begin{array}{l} 1\\ e^{-0.12\left(\theta -15\right)}\\ 0 \end{array}\right.\begin{array}{c} 5^{\circ }\leq \theta \leq 15^{\circ }\\ 15^{\circ }\leq \theta \leq 25^{\circ }\\ \theta \geq 25^{\circ } \end{array}$$

Areas where $\theta \geq 25^{\circ }$ were classified as high geological hazard risk zones.

Rock stratum stability was quantified using the Terrain Ruggedness Index (TRI) proposed by Riley et al. (1999) [22].

(2) $$TRI=\sqrt{\sum_{i=1}^{8}{\left(H_{i}-\bar{H}\right)}^{2}}$$

**Figure 4:**
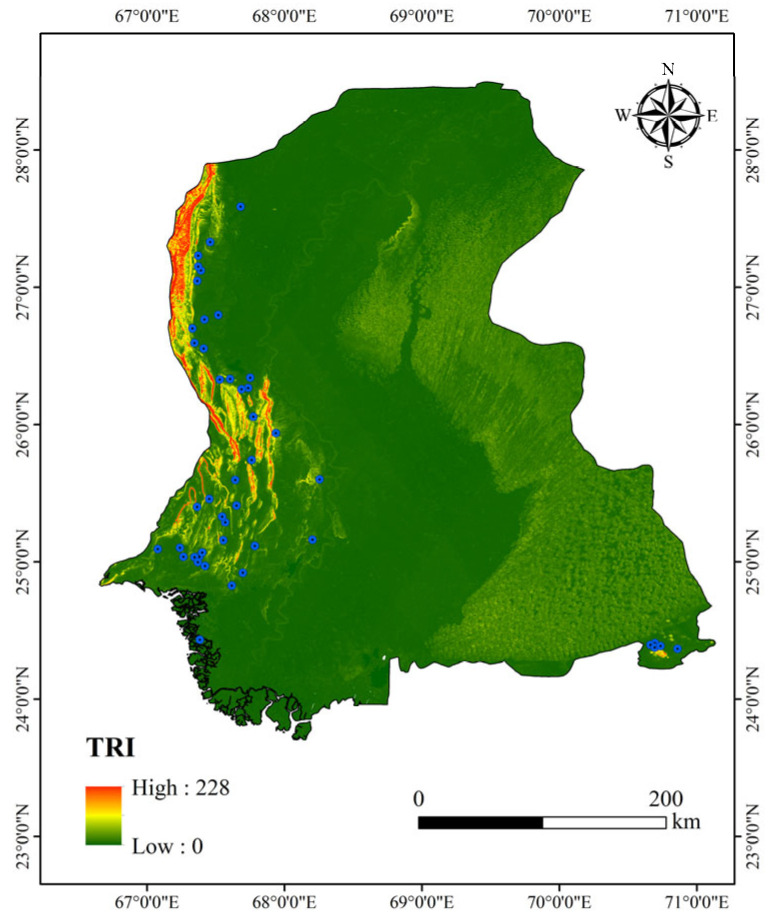
TRI of Sindh Region.

The TRI aids in excluding geologically unstable areas, as high ruggedness zones (*TRI* > 50) are often associated with fault activity, as shown in Figure 4. *TRI* ≤ 30 was set for screening stable dam foundations. Notably, the threshold of *TRI* ≤ 30 for screening stable dam foundations is not a fixed universal value but a dynamically determined parameter based on the statistical characteristics of existing dam samples in the study area. Specifically, through statistical analysis of TRI values from verified dam sites in Sindh Province, we found that total of these stable dam foundations exhibit TRI values ranging from 0 to 30, with the maximum TRI value among them being 29.7. This threshold was thus set to maximize coverage of real stable dam sites while minimizing inclusion of geologically risky areas.

#### 2.2.2. Hydrological Data

This study utilizes the HydroSHEDS global hydrological dataset released by the World Wide Fund for Nature (WWF) [23]. The 15 arc-second resolution (approximately 450 m) Flow Accumulation (FAC) raster data was selected as the foundational hydrological parameter. This dataset is specifically designed for hydrological modeling and water resource management at the watershed scale.

**Figure 5:**
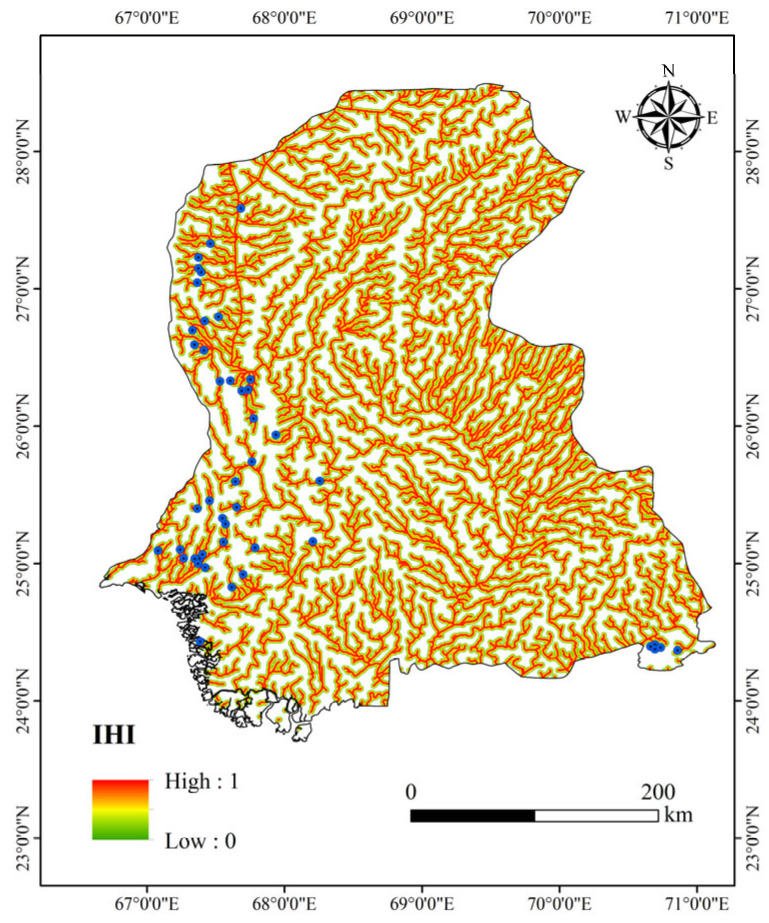
IHI of Sindh Region.

The association between dams and hydrological features manifests in factors like runoff volume and river distance. Grounded in the hydrological “source-sink” theory [24], this study proposes an adaptive hydrological threshold extraction method based on quantile analysis. By calculating the 75th percentile (i.e., pixels with FAC ≥ Q3) of the flow accumulation values within the study area, this method dynamically distinguishes seasonal runoff channels from perennial mainstem rivers, resulting in a comprehensive hydrological index (IHI), as shown in Figure 5. Compared to traditional fixed-threshold methods (e.g., FAC > 5000), this approach effectively mitigates the impact of regional hydrological heterogeneity. It specifically targets river segments exhibiting stable runoff characteristics, which are frequent locations for dam construction due to their potential for water resource regulation. This method adaptively excludes seasonal streams or tributaries, focusing instead on perennial river reaches with stable flow. These areas are more likely to host dams due to water resource management needs.

To quantify the topological relationship between dam site selection and the river network, this study developed a spatial decay function to generate a continuous hydrological factor: First, the shortest Euclidean distance (Ed) from each pixel to the nearest mainstem river was calculated using the Minkowski distance algorithm [25]. Subsequently, a distance decay index was established through linear normalization:

(3) $$H=\max\left(0,1-\left(\frac{Ed}{D_{max}}\right)\right)$$

where *D_max_* represents the maximum influence threshold (2000 m), and *H* ∈ [0, 1] characterizes spatial suitability. *H* = 1 indicates the river channel itself (*Ed* = 0), corresponding to the core potential zone for dam construction. *H* = 0.5 denotes the river buffer zone (*Ed* = 1000 m), while *H* → 0 represents the outer edge of the river’s influence domain (*Ed* ≥ 2000 m).

This continuous feature captures the nonlinear spatial relationship between dams and river channels through gradient variation, demonstrating higher sensitivity compared to traditional binary buffer methods.

#### 2.2.3. OSM Hydrographic Vector Data

OpenStreetMap (OSM) is an open-source mapping platform collaboratively created and maintained by global users, allowing real-time editing and updates based on field conditions. Its water vector data undergoes dynamic updates via a crowdsourced model, maintaining high timeliness and open-sharing characteristics. Regarding data types, OSM water data employs vector formats to precisely represent diverse waterbody features. Areal waterbodies (e.g., lakes, reservoirs) contain geometric boundaries and area attributes, while linear waterbodies (e.g., rivers) incorporate hydrological parameters such as flow direction and width. Dynamic attributes distinguish perennial from seasonal waterbodies via tags, enabling the selection of perennial stable water areas.

In this study, dam locations typically occur adjacent to waterbodies, excluding areas like bare soil or vegetation cover. Utilizing OSM water data from April 2025, we obtained the spatial distribution of perennial waterbodies. The use of April 2025 data ensures the incorporation of the most recent updates, reflecting current field conditions. Additionally, this period corresponds to a time of lower rainfall and stable climate in the study region, facilitating the identification of perennial stable waterbodies by minimizing seasonal variations. This provided spatial constraints for high-probability areas, further narrowing candidate areas and establishing geographical prior conditions for dam site selection. Although OSM data benefits from real-time global user contributions and community-based validation updates, to further verify its accuracy, we randomly selected 20 representative sites across major terrain categories (mountainous areas, river valleys, plains) within the study area, covering key water body types (rivers, reservoirs, irrigation canals). Comparison with high-resolution imagery (Google Earth, 0.3-m resolution) through random sampling, over 95% of areas labeled as “permanent water bodies” in OSM correspond to actual water extents in the imagery. This meets the constraint requirement for excluding non-water areas.

#### 2.2.4. Dam Image Sample Set

The publicly available river barrier image dataset is the RBOD [26], containing five categories (weirs, dams, groynes, sluices, locks). To focus on our detection target, we extracted the dam category exclusively, yielding 2386 dam instances. To better align with the vegetation-sparse topographic background of Pakistan and enhance the model’s generalization capability within the study area, we integrated global open-access dam spatial databases (including records for Sindh Province from GDW and GRanD) and collected officially recorded dam location data from the website of the Irrigation Department of Sindh, Pakistan (https://irrigation.sindh.gov.pk/gis/gismaps, accessed on 3 March 2025). Precise spatial locations were extracted, resulting in a final set of 2450 dam instances. Research by Soares et al. (2020) indicates that image augmentation effectively improves the accuracy of deep learning models [27]. This study implemented a random augmentation strategy on data labeled as dams to enhance model generalization, employing augmentation across three dimensions. At the geometric transformation level, images were randomly rotated by 90°, 180°, and 270°, and subjected to horizontal and vertical flipping, simulating dam imaging characteristics under varying perspectives and scales. For color enhancement, adjustments to brightness and contrast simulated image effects under diverse lighting and atmospheric conditions (e.g., cloudy days), accentuating the contrast between dams and their background. Ultimately, the ratio of original samples to augmented samples is 1:4. Gaussian blur [28] and Gaussian noise addition [29] were also introduced to simulate interference factors present in actual remote sensing imagery. It is emphasized that image augmentation was applied solely to the model training sample set and did not involve the original high-resolution remote sensing imagery used during the dam identification phase. Ultimately, 12,250 dam image samples were obtained, forming a robust dataset for model training and evaluation. To ensure the reliability and generalization of the trained YOLOv11 model, the dataset was partitioned using a standard 7:1:2 ratio for training, validation, and testing, respectively. The specific numbers are shown in Table 1.

### 2.3. Methodologies

**Figure 6:**
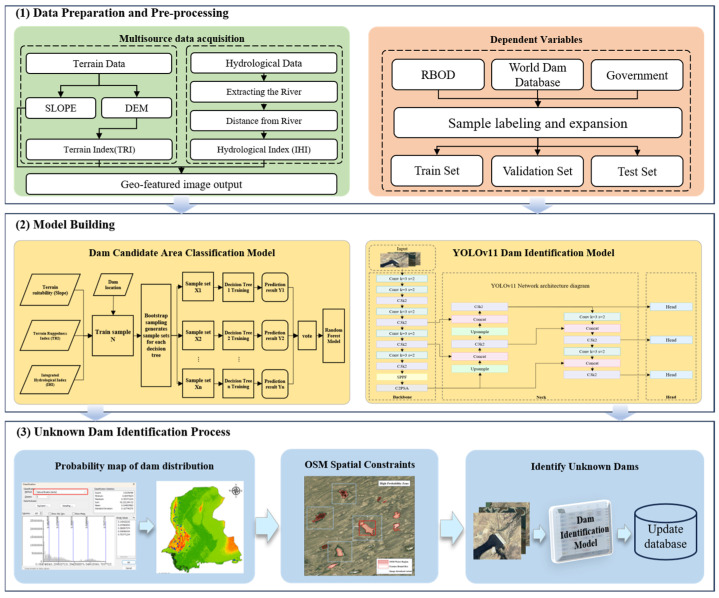
The hybrid framework of Dam Detection.

The proposed dam identification framework comprises three primary steps: (1) Generating a dam distribution probability map by fusing multi-source remote sensing data using the Random Forest algorithm; (2) Spatially constraining dam candidate areas using OSM water body data. (3) Identifying dams within candidate areas that were not initially captured, using a deep learning-based image recognition model. Within this framework: The first step utilizes multi-source remote sensing data, trained on verified dam locations, to identify high-probability zones as dam candidate areas. This provides reliable spatial constraints over large areas regarding potential dam presence. Subsequently, OSM water body vector data is applied to exclude non-water areas within the candidate areas obtained in the previous step, further refining the target areas. Finally, the YOLO image recognition algorithm processes imagery within these refined candidate areas to extract precise dam locations, thereby enhancing the original database. This model, built upon the dam dataset constructed in this study, achieves rapid inference with minimal computational demands and reliable feature representation while maintaining robust performance, as illustrated in Figure 6.

#### 2.3.1. Dam Candidate Area Classification Model

**Figure 7:**
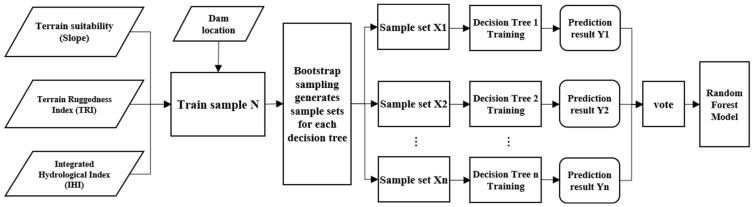
Flowchart of Classification Model for Dam Candidate Areas.

Random Forest (RF) [30], an ensemble learning model, was proposed by statisticians Leo Breiman and Adele Cutler. This technique performs classification or regression tasks by constructing multiple decision trees in parallel. Its core innovation lies in introducing a dual randomization mechanism: firstly, using Bootstrap sampling to create diverse training subsets [31], and secondly, employing random feature subspace selection to reduce inter-tree correlation. Final predictions are generated through majority voting (for classification tasks) or averaging (for regression tasks), effectively mitigating model overfitting risks. The flowchart is shown in Figure 7.

Compared to traditional classification models (e.g., K-Nearest Neighbors [32]), RF demonstrates significant advantages in the following aspects: First, it offers higher efficiency and robustness. The Bagging ensemble strategy enhances computational efficiency, reducing training time per tree by approximately 40%, while maintaining performance in high-noise data environments. Second, it supports parallel processing of multi-source geographical features, preventing the curse of dimensionality. Third, it effectively models nonlinear relationships, capturing complex interactions between terrain and hydrological factors. In this study, three dam location influencing factors were selected as independent variables, with dam presence probability as the dependent variable. The Random Forest model effectively captured the complex relationships between geographical factors and dam presence, establishing a robust predictive model.

After generating the dam probability distribution map using the random forest model, it is essential to quantitatively evaluate the reliability of the classification results using multiple metrics and validate the scientific basis for candidate region delineation. This study employs Accuracy, Precision, Recall, and the F1-Score to assess the classification performance of the random forest model. The corresponding formulas are as follows.

(4) $$Accuracy=\frac{TP+TN}{TP+TN+FP+FN}$$

(5) $$Precision=\frac{TP}{TP+FP}$$

(6) $$Recall=\frac{TP}{TP+FN}$$

(7) $$F1-Score=\frac{2\times Precision\times Recall}{Precision+Recall}$$

where *TP*, *FP*, and *FN* denote true positive, false positive, and false negative, respectively.

#### 2.3.2. Screening Strategy for Dam Candidate Area Imagery

The dam distribution probability map generated in the previous step initially delineated potential dam locations. Considering the typical placement of dams on rivers, lakes, reservoirs, and other water bodies, barren soils and vegetated areas were subsequently excluded. Given its advantages of multiscale coverage, dynamic updates, and rich attributes, OSM water vector data were introduced to provide spatial constraints. This data clearly reflects the presence of various water bodies. By overlaying the high-probability dam distribution areas with the OSM water vector data, potential dam sites were identified at the intersections of high-probability patches and water body polygons. This secondary delineation further narrowed down the target candidate regions.

**Figure 8:**
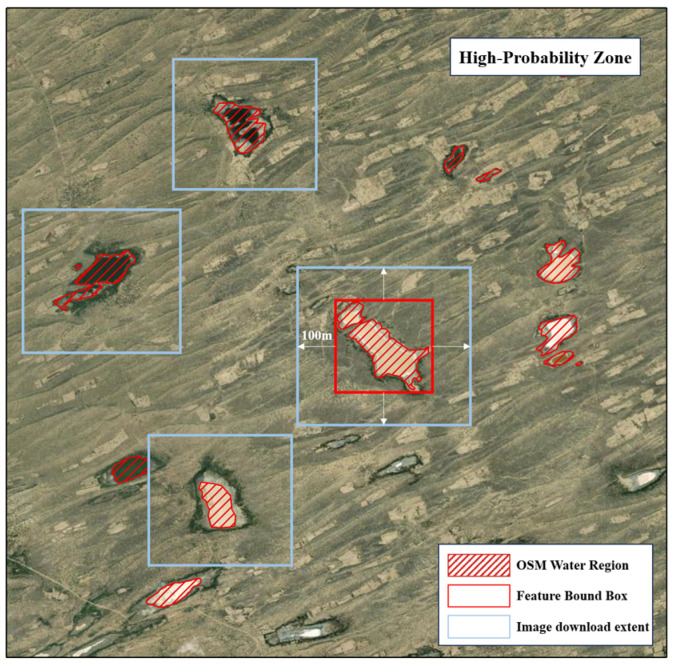
High Probability Area Image Download Strategy Schematic Diagram.

Figure 8 illustrates the image download strategy for the high-probability dam candidate regions. Multisource high-resolution remote sensing imagery was acquired via the Google Earth platform. Download areas were centered on the final candidate regions and expanded outward sufficiently to ensure complete coverage of the potential dam locations. Google Earth imagery offers a maximum spatial resolution of 0.3 m, which reveals dam structure details clearly. This high-resolution data facilitates the subsequent identification and extraction of dams within the candidate regions.

#### 2.3.3. YOLOv11 Dam Identification Model

**Figure 9:**
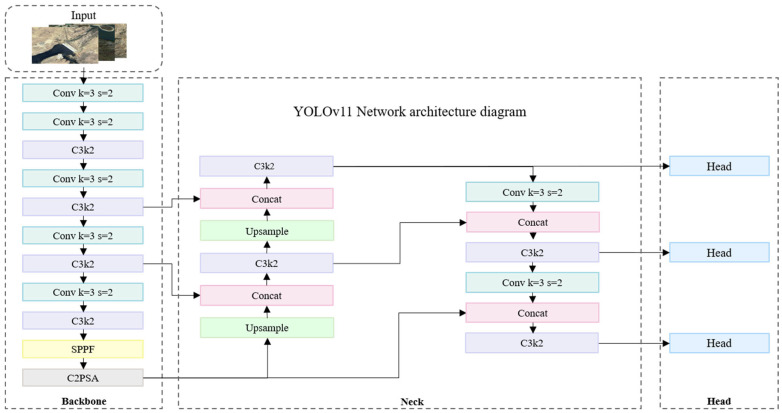
YOLOv11 Network architecture diagram.

The YOLOv11 (You Only Look Once Version 11) framework employed in this study represents the latest iteration of the YOLO object detection series, with its overall architecture illustrated in the Figure 9. Compared to YOLOv8, this model retains the computational efficiency inherent in single-stage detectors while introducing innovative enhancements to its network architecture, feature fusion mechanisms, and loss function design. These improvements substantially boost detection accuracy and processing speed in complex scenarios [33]. In addition, in river barrier detection based on the RBOD dataset (the same source used for dam samples in this study), Reference [26] reported that the YOLOv8 series significantly outperformed traditional oriented detection models such as Oriented R-CNN and Rotated Faster R-CNN, with YOLOv8x-OBB achieving an mAP@0.5 of 0.82. This finding established YOLOv8 as a strong baseline for detecting linear hydraulic structures in high-resolution imagery. According to official benchmark evaluations, YOLOv11 further extends the strengths of YOLOv8, offering superior detection accuracy, and especially for small-scale targets in cluttered backgrounds while simultaneously accelerating inference. Building upon this model, we trained our previously constructed dam dataset to develop a specialized dam detection model.

During training, we implemented the Stochastic Gradient Descent (SGD) optimizer with an initial learning rate of 0.001 and momentum of 0.9. To enhance generalization and mitigate overfitting, the learning rate was reduced by a factor of 10 every 10 epochs. All experiments were executed using PyTorch 2.60 on a computational platform equipped with an NVIDIA RTX 4090 GPU (24 GB) and an Intel Xeon Gold 6226R CPU. The total training time for the 12,250 samples was approximately 6 h, with a peak GPU memory usage of 18.2 GB. During the inference phase, the processing time for a single image (2560 × 2560 pixels) was 0.05 s. The training was repeated iteratively until validation scores—such as precision, recall, F1-score, and mAP50—stabilized and the loss function converged to a satisfactory level.

## 3. Results and Analysis

### 3.1. Dam Existence Probability Distribution Results

**Figure 10:**
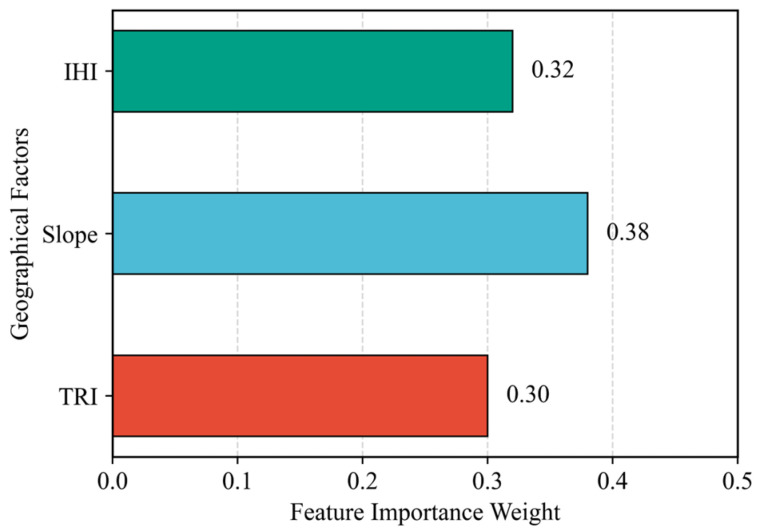
Feature Importance.

The location of dams is closely associated with geographic and hydrological elements. By integrating the influencing factors mentioned above—namely, Slope, Terrain Ruggedness Index (TRI), and the Integrated Hydrological Index (IHI)—we overlaid the feature layers. The Random Forest algorithm was employed to capture features correlated with dam presence, resulting in a dam distribution probability map. To thoroughly analyze the impact of various indicators on dam existence probability, this study recorded the weight values assigned by the Random Forest algorithm during the construction of the full-sample model to evaluate variable importance, with the results presented in Figure 10. Among these factors, slope was assigned the highest weight as a core factor, since it directly determines the feasibility of foundational engineering for dams and was explicitly linked to dam existence probability via a stability classification function. The comprehensive hydrological index, which quantifies the spatial relationship between potential dam sites and river networks based on source-sink theory, received the second highest weight due to its role in determining the value of water resource regulatory functions. In contrast, TRI, which assists in screening geologically stable areas by assessing bedrock stability, exerted a more indirect influence on dam probability and was consequently assigned the lowest weight. However, The Random Forest model outputs continuous probabilities (ranging from 0 to 1) for the potential dam areas. Dynamic threshold segmentation was applied to convert the result into discrete probabilities indicating high or low likelihood of potential dam areas. This provides spatial constraints for the subsequent refined detection using the YOLOv11 model.

**Figure 11:**
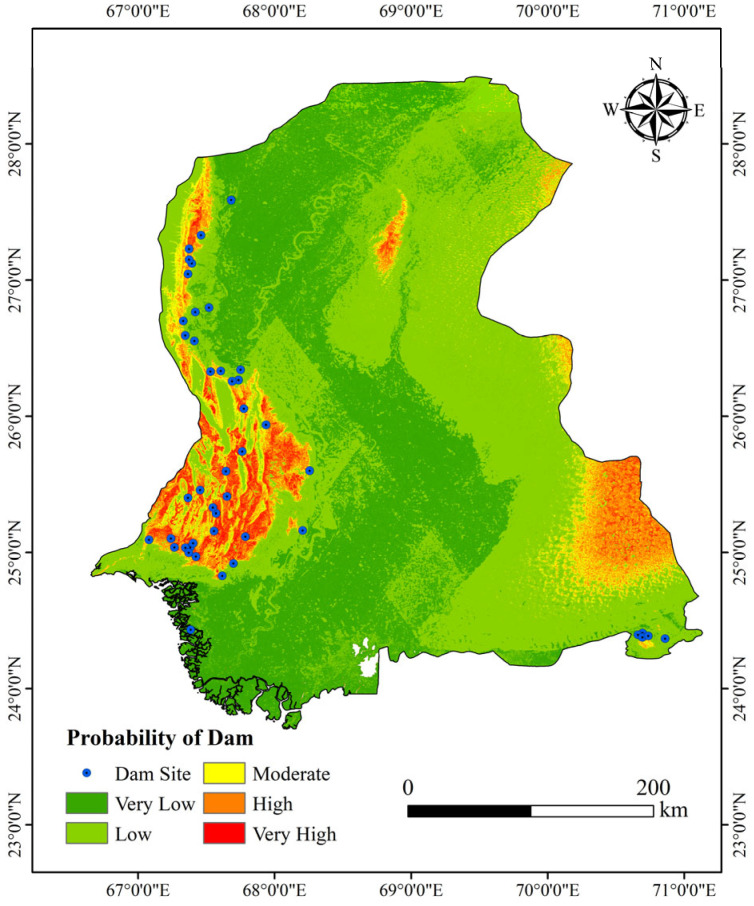
The dam probability distribution map of Sindh Province.

Figure 11 shows the dam probability distribution map for Sindh Province, Pakistan. Using the Natural Breaks Classification (Jenks) method in ArcGIS, the dam existence probability was classified into five categories: Low, Moderately Low, Moderate, Moderately High, and High. This data-driven approach, which iteratively minimizes within-class variance and maximizes between-class variance, effectively reduced the subjectivity associated with manual threshold setting throughout the process. Central and eastern Sindh, encompassing the Lower Indus Plain and Indus River Delta, predominantly exhibit Low and Medium-Low probabilities. Medium and Medium-High probabilities are observed in north-central and southeastern regions. The western part, characterized by the Kirthar Mountains and hills, shows predominantly Medium-High and High probabilities, displaying a linear distribution pattern. The significant elevation drop in this area creates a water level differential, which is conducive to dam construction. Overall, the spatial pattern of dam probabilities aligns with known dam locations. Dams are primarily concentrated in western Sindh, showing high overlap with rivers, consistent with the characteristic placement of dams near water bodies.

### 3.2. Dam Existence Probability Distribution Accuracy Analysis

**Figure 12:**
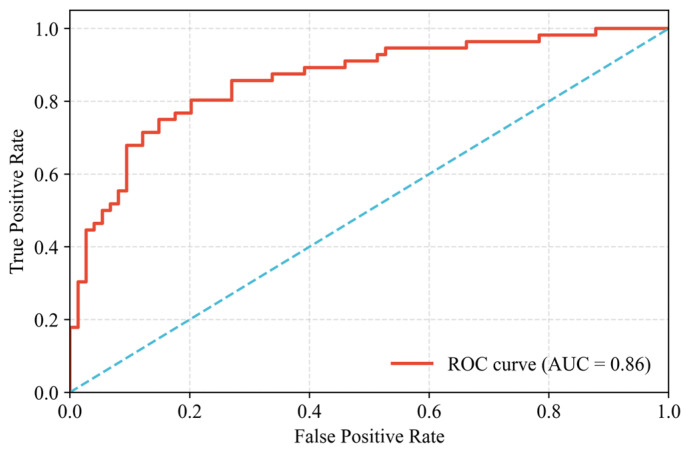
ROC curve of the dam probability model’s classification.

The model attained an accuracy of 0.91, signifying a high proportion of correct classifications regarding dam presence or absence. Precision was measured at 0.90, denoting a low incidence of falsely identifying dams where none exist. Conversely, the recall score of 0.76 indicates that a substantial portion of actual dams (24%) remained undetected, pointing to a critical area for model enhancement to minimize missed detections. Further evaluation using the Receiver Operating Characteristic (ROC) curve yielded an Area Under the Curve (AUC) value of 0.86, as shown in Figure 12. Typically, an Area Under the Curve (AUC) value in the range of (0.7, 0.9] indicates that the model has good predictive accuracy [34]. This AUC score indicates a good level of discriminatory power for the model in distinguishing between dam presence and absence across all possible classification thresholds. While the model provides preliminary effectiveness in identifying dam distribution probability, its insufficient recall may overlook actual dams despite the relatively strong overall discriminative ability reflected in the AUC.

### 3.3. Accuracy Evaluation of Dam Recognition Model

To thoroughly evaluate the performance advantages of the YOLOv11 model in dam image recognition tasks, this study conducted a comprehensive comparative analysis against multiple mainstream object detection models using our custom-built dataset. The compared models cover representative single-stage and two-stage detectors, including YOLOv8, R-CNN, Faster R-CNN, and RetinaNet. Detailed performance comparison results are presented in Table 2.

As shown in Table 2, YOLOv11 demonstrates significant performance advantages on our custom dam image dataset. Compared with other mainstream object detection models, YOLOv11 achieves the highest scores across all metrics: Precision (0.85), Recall (0.83), F1_Score (0.84), and mAP50 (0.85), outperforming representative single-stage and two-stage detectors including YOLOv8, R-CNN, Faster R-CNN, and RetinaNet. These results indicate that YOLOv11 not only exhibits stronger accuracy but also achieves a better balance in detection comprehensiveness and stability. Notably, its leading performance on the comprehensive and representative metrics F1_Score and mAP50 further validates YOLOv11’s enhanced detection capability and practical applicability in complex backgrounds such as dam structures.

**Figure 13:**
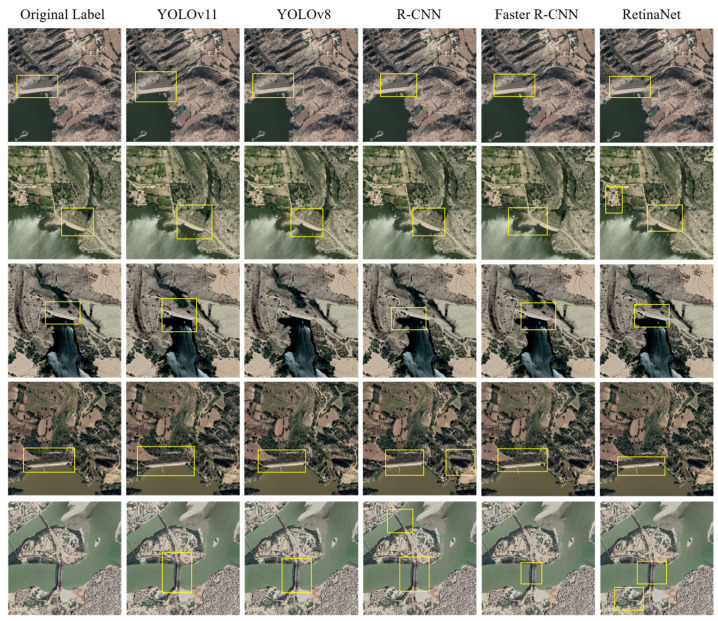
Sample detection results from various object detection algorithms, including YOLOv11, YOLOv8, R-CNN, Faster R-CNN, RetinaNet.

Figure 13 further confirms the superior performance of YOLOv11 in dam detection. Significant differences exist in the detection results of different models for the same target. For instance, YOLOv11 consistently successfully and accurately delineated complete dam regions in most images, demonstrating precise bounding box localization with fewer instances of missed or false detections. In contrast, other models such as R-CNN, RetinaNet, and YOLOv8 exhibited notable limitations, including boundary shifts, incomplete bounding boxes, false positives, and even missed detections. Although Faster R-CNN delivered relatively stable overall performance, it still suffered from inaccurate localization of slender structures, particularly in images with complex backgrounds and significant water interference, indicating a lack of robustness. However, experiments revealed that the YOLOv11 model still has issues, as it tends to misclassify linear structures similar to bridges as dams (as shown in the last row of Figure 13), thereby increasing the misidentification rate of target elements. We will focus on optimizing this issue in future research.

The overall results demonstrate that YOLOv11 maintains consistent detection performance even in images with clear structures and significant illumination variations. This indicates its strong adaptability to scale and viewpoint changes. Such an advantage is particularly crucial in the complex and variable environments typical of remote sensing scenarios. Therefore, in practical dam inspection tasks, YOLOv11 can provide not only higher accuracy but also more reliable extraction and usability of target regions. This is the primary reason for selecting YOLOv11 as the foundational model in this study.

### 3.4. Comparative Analysis of the Hybrid Framework

#### 3.4.1. Performance of YOLOv11 Before and After RF Filtering

To quantitatively verify the efficiency improvement in the hybrid framework, we compared the detection performance of the YOLOv11 model in two scenarios: without RF filtering (direct full-image detection) and with RF filtering (detection within RF-delineated candidate areas). The comparison focuses on key metrics such as precision, recall, F1-score, mAP50, and false positives, as shown in Table 3.

With RF filtering, precision increased by 27% (from 0.67 to 0.85) and mAP50 improved by 33% (from 0.64 to 0.85). This is because RF filtering effectively narrows the detection scope to high-probability areas, reducing interference from non-dam backgrounds that are prone to false detection. False positives decreased by 76% (from 84 to 31), confirming that RF-based candidate area delineation significantly reduces redundant computations in non-target regions—addressing the “low efficiency in full-image processing” limitation of existing methods.

#### 3.4.2. Novelty of the Hybrid Framework Compared to Existing Methods

The proposed RF-YOLOv11 hybrid framework exhibits distinct innovations compared to existing YOLO-based or hybrid detection systems, addressing critical limitations in prior research. First, it integrates multi-source geographic factor analysis by using the RF algorithm to fuse slope, TRI, and IHI, generating a data-driven probability map that quantifies the nonlinear relationship between geographic features and dam presence, reducing reliance on empirical thresholds and enhancing candidate area delineation. Second, it employs a two-stage spatial constraint approach, where RF initially narrows high-probability areas and OSM water body data further excludes non-water regions, reducing the detection area by approximately 82% and significantly lowering computational costs while maintaining accuracy. The method proposed in this study is compared with existing methods as shown in Table 4.

These comparisons highlight that the proposed framework advances dam detection by integrating geographic intelligence, optimizing detection scope, and enhancing regional adaptability—effectively addressing the limitations of existing methods.

### 3.5. Dam Identification Results and Analysis

**Figure 14:**
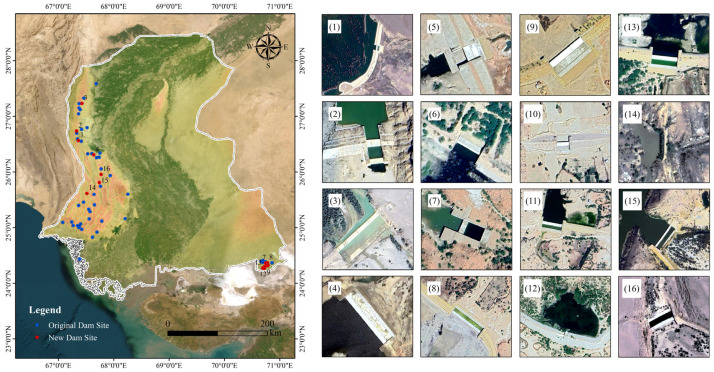
Sindh Province Dam Identification Results. The main figure on the left illustrates the newly identified dam sites compared to the original sites. The corresponding subfigures (**1**)–(**16**) on the right present detailed imagery of each identified dam.

During the identification of dam candidate areas from imagery, this study successfully detected 16 dam entities not recorded in the official database, as shown in Figure 14. Spatially, the newly detected dams exhibit distinct geographic distribution patterns. Seven dams are located in western Sindh Province, clustered where the Kirthar Mountains meet river tributaries. This area features significant topographic relief, aligning with typical dam siting requirements for utilizing elevation differences and water bodies. The dams identified in this area primarily form small reservoirs by intercepting mountain runoff, serving both flood control (regulating seasonal flash floods) and supplementary irrigation functions. However, complex terrain and poor accessibility hindered comprehensive coverage by traditional surveys. Nine dams were identified in the southeastern region, primarily at junctions between irrigation branch canals and seasonal rivers. These serve decentralized irrigation needs for cotton and rice cultivation, reflecting strong local demand for agricultural water infrastructure, which corresponds with actual conditions. All identified dams are small- to medium-sized, situated on small reservoirs or rivers, explaining their omission from prior statistics. For instance, some small and medium dams detected in the southeast were missed in past surveys due to their remote locations, sparse population, and lack of nearby road access. Small irrigation dams on river branches were excluded from existing inventories due to their limited scale. Although the upper part of the southeast region showed medium-to-high probability, OSM water body data indicated no reservoirs or lakes present, so it was excluded from the final candidate areas, and no dams were identified there.

From an applied perspective, the model, guided by potential area information from the probability map and leveraging detailed features from high-resolution imagery, successfully captured targets easily overlooked by conventional methods. This significantly improves the efficiency of spatial dam database compilation. These findings provide crucial supplemental information for regional dam resource management. Multi-temporal satellite image comparisons can determine the construction period, functional purpose, and safety status of these dams, offering foundational data support for regional water resource management and disaster early warning. These results also demonstrate the significant advantage of remote sensing imagery combined with deep learning detection methods in filling gaps in geographic information and aiding resource inventories. To further enhance the practical relevance of these findings, integrating the detection results into real-world GIS applications and validation workflows is critical. The identified dam coordinates (including the 16 newly detected small- and medium-sized dams) can be exported in vector formats to supplement regional GIS databases, such as the GIS system of Sindh Province’s Irrigation Department, enabling spatial overlay analysis with existing hydrological facility layers, terrain data (DEM), and hydrological networks. Within GIS platforms, a visualization module linking “detection results, high-resolution imagery, and geographic factors” can be constructed, allowing managers to intuitively verify the alignment between dam distribution and topographic/hydrological features.

## 4. Discussion

Although this study achieved certain results, it remains constrained by limitations in data, methodology, and technology, with the following areas requiring further improvement:

The dataset exhibits limitations and bias. The current dataset has relatively low dimensionality, with all imagery sourced solely from Google Earth and primarily at sub-meter resolution. This results in the entire workflow being highly dependent on high-resolution imagery. Furthermore, the dataset constructed in this study is based on the RBOD dataset (a global dataset with a disproportionately high representation of warm temperate zones). The dense vegetation in these regions enhances the feature discrimination between dams and their background environments. Although we supplemented it with some local dam imagery from Pakistan, the quantity was limited, resulting in its continued low proportion within the overall dataset. The background environmental characteristics in Pakistan make dams harder to distinguish from their surroundings. Consequently, the dataset lacks sufficient samples of local Pakistani dams and exhibits insufficient diversity in scene coverage.

The classification model does not comprehensively consider all relevant factors. In determining the probability of dam presence, the current study only integrates geographical and hydrological factors. While good classification accuracy was achieved, it fails to adequately incorporate other key factors influencing dam site selection, such as climatic elements (e.g., precipitation, temperature) and socio-economic elements (e.g., economic activity, agriculture). Therefore, the model’s discriminative basis remains incomplete.

This study implemented no specific modifications to the YOLOv11 recognition model for dams. Although good recognition performance was attained, instances of misidentification still occur during the recognition process. For example, the model misidentifies dam-like structures such as bridges as dams. These objects share geometric similarities with dams and can become significant interference factors in dam recognition.

During validation in Sindh Province, the framework demonstrated effective identification capabilities for small- to medium-sized dams. However, its generalizability across diverse geographical regions remains challenging. Sindh Province is predominantly characterized by the flat Indus River Plain, featuring relatively gentle terrain and stable water body morphology. If extended to areas with different topographies, the spatial weighting of geographical factors may undergo significant changes. Hydrological indices in arid regions rely on the stability of perennial rivers. When applied to humid regions, however, the frequent channel migration of seasonal rivers could reduce the delineation accuracy of potential dam sites identified by the RF model. Furthermore, this study primarily targets small- to medium-sized irrigation and flood control dams. For large-scale hydraulic structures, model specialization may be required to optimize performance under complex scenarios.

## 5. Conclusions

This study proposes a “two-stage collaborative” framework for dam identification, successfully achieving efficient dam detection in Sindh Province, Pakistan. First, a dam probability distribution map (F1-Score 0.83) was constructed by integrating topographic and hydrological factors. This map was combined with OSM water body data to precisely delineate high-probability candidate areas, significantly reducing the target scope. Subsequently, leveraging a multi-source enhanced sample library, a lightweight YOLOv11 model (mAP 0.85) was trained. This model efficiently detected 16 unrecorded small-to-medium dams in complex environments. The results demonstrate that this method offers significant technical advantages and application value in the field of dam extraction from remote sensing imagery, providing reliable technical means and a data foundation for water resource management and disaster early warning.

Future research will focus on constructing a more balanced and representative multi-source remote sensing dataset. This will significantly increase the sample proportion for key regions like Pakistan and diverse environmental scenarios while reducing reliance on single high-resolution imagery. Concurrently, climate factors (e.g., precipitation, temperature) and socio-economic factors (e.g., economy, agriculture) will be systematically integrated to enhance the comprehensiveness and discriminant basis of the dam existence probability classification model. Furthermore, the object detection model will be specifically optimized (e.g., by introducing attention mechanisms and strengthening hard negative sample learning) to effectively distinguish dams from geometrically similar structures like bridges. These improvements aim to comprehensively enhance identification accuracy and generalization capability.

## Figures and Tables

**Table 1:** The distribution of training, validation and test datasets.

Class	Train	Validation	Test	Total
Dam	8575	1225	2450	12,250

**Table 2:** Comparison of the different models.

Models	Precision	Recall	F1_Score	mAP50
YOLOv8	0.81	0.78	0.79	0.78
R-CNN	0.79	0.72	0.75	0.64
Faster R-CNN	0.81	0.76	0.78	0.67
RetinaNet	0.76	0.70	0.73	0.59
YOLOv11	0.85	0.83	0.84	0.85

**Table 3:** Comparison of YOLOv11 Before and After RF Filtering.

Scenario	Precision	Recall	F1_Score	mAP50	False Positives
YOLOv11	0.67	0.70	0.68	0.64	130
RF-YOLOv11	0.85	0.83	0.84	0.85	31

**Table 4:** Hybrid Framework Compared to Existing Methods.

Method	Precision	Recall	F1_Score	Computational Efficiency
YOLOv5x-ViT-BiFPN + cropping [18]	0.84	0.82	0.80	Low (full-image scanning)
E-YOLO + NDWI constraint [19]	0.691	0.884	0.776	Low (full-image scanning)
This study	0.85	0.83	0.84	High (82% area reduction)

## Data Availability

The original contributions presented in this study are included in the article. Further inquiries can be directed to the corresponding author.
